# Genome Sequence of a Staphylococcus epidermidis Strain (GTH12) Associated with Candida albicans SC5314 Cultured under Hypoxia at 37°C in Glycerol for 12 Weeks

**DOI:** 10.1128/genomeA.00533-18

**Published:** 2018-06-21

**Authors:** Danielle do Carmo Ferreira Bruno, Thais Fernanda Bartelli, Marcelo R. S. Briones

**Affiliations:** a Department of Microbiology, Immunology and Parasitology, Laboratory of Evolutionary Genomics and Biocomplexity, Universidade Federal de São Paulo, São Paulo, Brazil; b Department of Health Informatics, Universidade Federal de São Paulo, São Paulo, Brazil

## Abstract

Polymicrobial infections with mixed-species biofilms are important health problems because of increased antimicrobial resistance and worse patient outcomes than with monomicrobial infections. Here, we present the whole-genome sequence of Staphylococcus epidermidis strain GTH12, which was cocultured with the yeast Candida albicans SC5314 (generating C. albicans strain SC5314 GTH12), thus providing genomic information on polymicrobial infections.

## GENOME ANNOUNCEMENT

Nosocomial polymicrobial infections are serious health problems, especially in immunocompromised hosts that can be infected from their own microbiome or from contaminated medical devices, forming mixed-species biofilms that are associated with increased antimicrobial resistance and virulence. Commonly, polymicrobial infections are associated with coagulase-negative staphylococci (CNS), such as Staphylococcus epidermidis, as well as Candida species (1–3). Bloodstream infections caused by Candida albicans are associated with tissue invasion, and staphylococcal infections are believed to be facilitated by these bacterium-hypha associations once S. epidermidis can strongly adhere to C. albicans hyphal and yeast forms (1, 4–6). In this study, we identified an S. epidermidis strain (GTH12) cocultured with our C. albicans strain SC5314 (generating C. albicans strain SC5314 GTH12), whose genome was previously published (7). This polymicrobial culture was grown on a YPD plate (1% [wt/vol] yeast extract, 2% [wt/vol] peptone, 2% [wt/vol] dextrose, 2% [wt/vol] agar), and a single colony was used for continuous growth for 12 weeks on YPG broth (1% [wt/vol] yeast extract, 2% [wt/vol] peptone, 2% [wt/vol] glycerol) at 37°C under hypoxic conditions (5 to 15% oxygen level). Total DNA was extracted as described previously (8), and sequencing was carried out by the Illumina MiSeq 2 × 300-bp method in paired-end mode. The libraries were prepared with the TruSeq DNA version 2 Illumina kit according to the manufacturer’s technical specifications. The FastQC version 0.11.4 software (9) was used to evaluate sequencing quality. Trimming was performed with the CLC Genomics Workbench version 7.5.1 (Qiagen) software with a quality score limit of 0.005 and removal of 45 bp and 20 bp from the 5ʹ and 3ʹ ends, respectively, discarding reads smaller than 25 bp. Once the quality filters were approved, reads were simultaneously mapped to the fasta sequence available for the Staphylococcus epidermidis ATCC 12228 reference strain (GenBank accession number NC_004461) and strain SC5314 (A22-s07-m01-r57) (available at http://www.candidagenome.org/cgi-bin/genomeVersionHistory.pl).

The fraction of the S. epidermidis GTH12 genome sequenced was 0.93, with an average coverage of 223×, ranging from 0 to 9,653×.

### Accession number(s).

The S. epidermidis GTH12 sequence obtained in this study has been deposited in GenBank under accession number CP028282. Raw sequencing data are available from the NIH Sequence Read Archive (BioProject number PRJNA434386) under BioSample accession number SAMN08555375.
